# Supplementary figures and images for: USP22 overexpression fails to augment tumor formation in MMTV-ERBB2 mice but loss of function impacts MMTV promoter activity
Source: PLoS One. 2024 Jan 18;19(1):e0290837. doi: 10.1371/journal.pone.0290837 (PMC10796002; doi:10.1371/journal.pone.0290837)

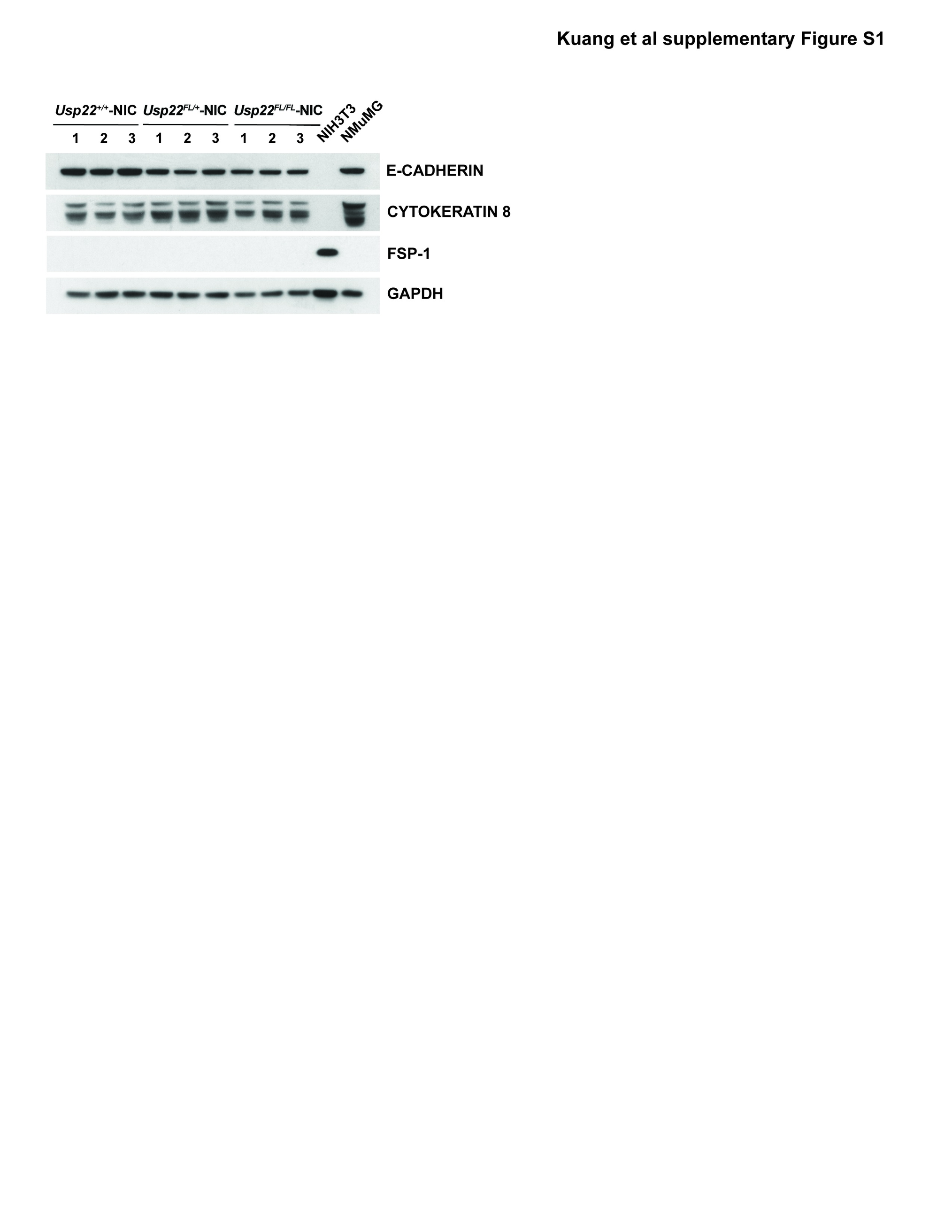

Supplement: S1 Fig — (TIF) [file pone.0290837.s001.tif]

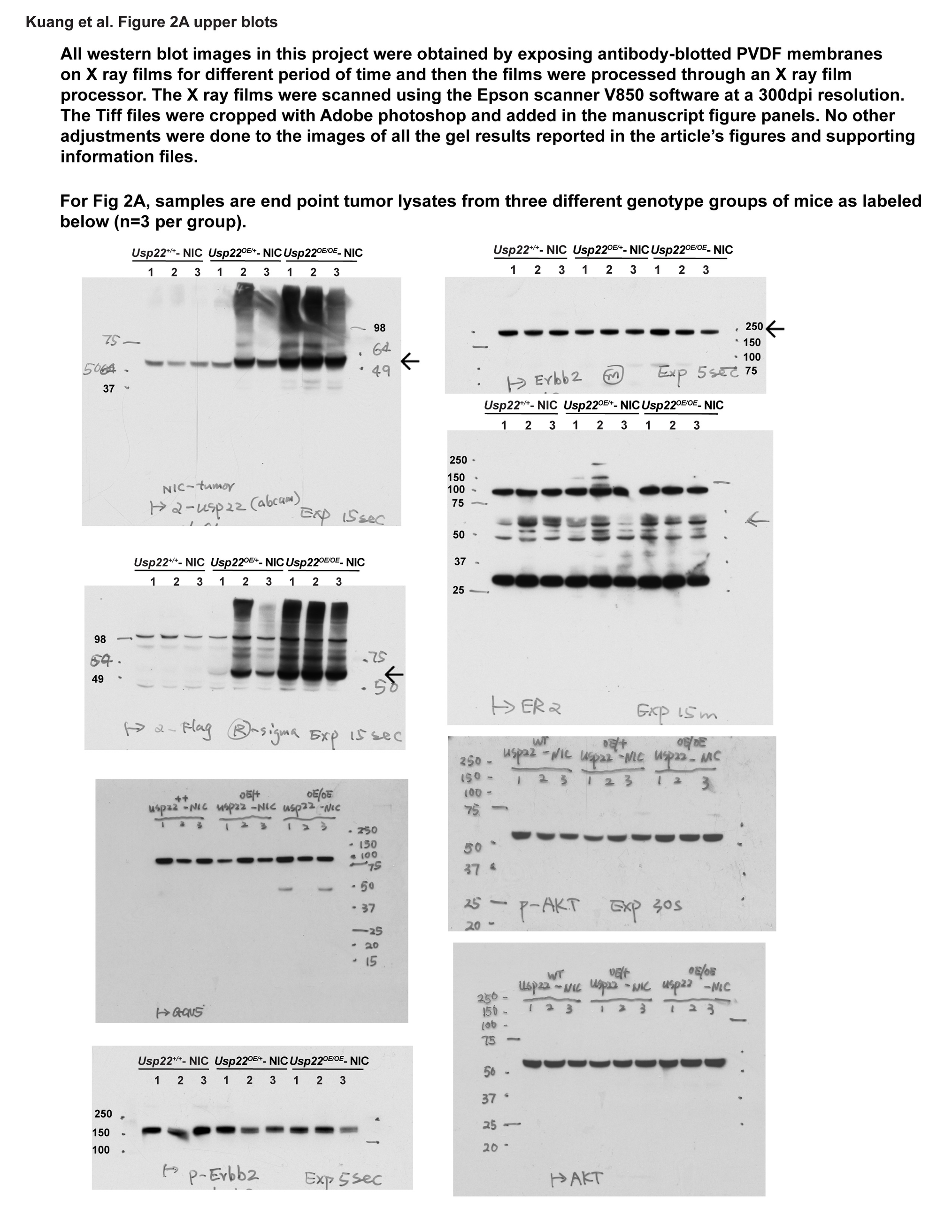

Supplement: S2 Fig — (TIF) [file pone.0290837.s002.tif]

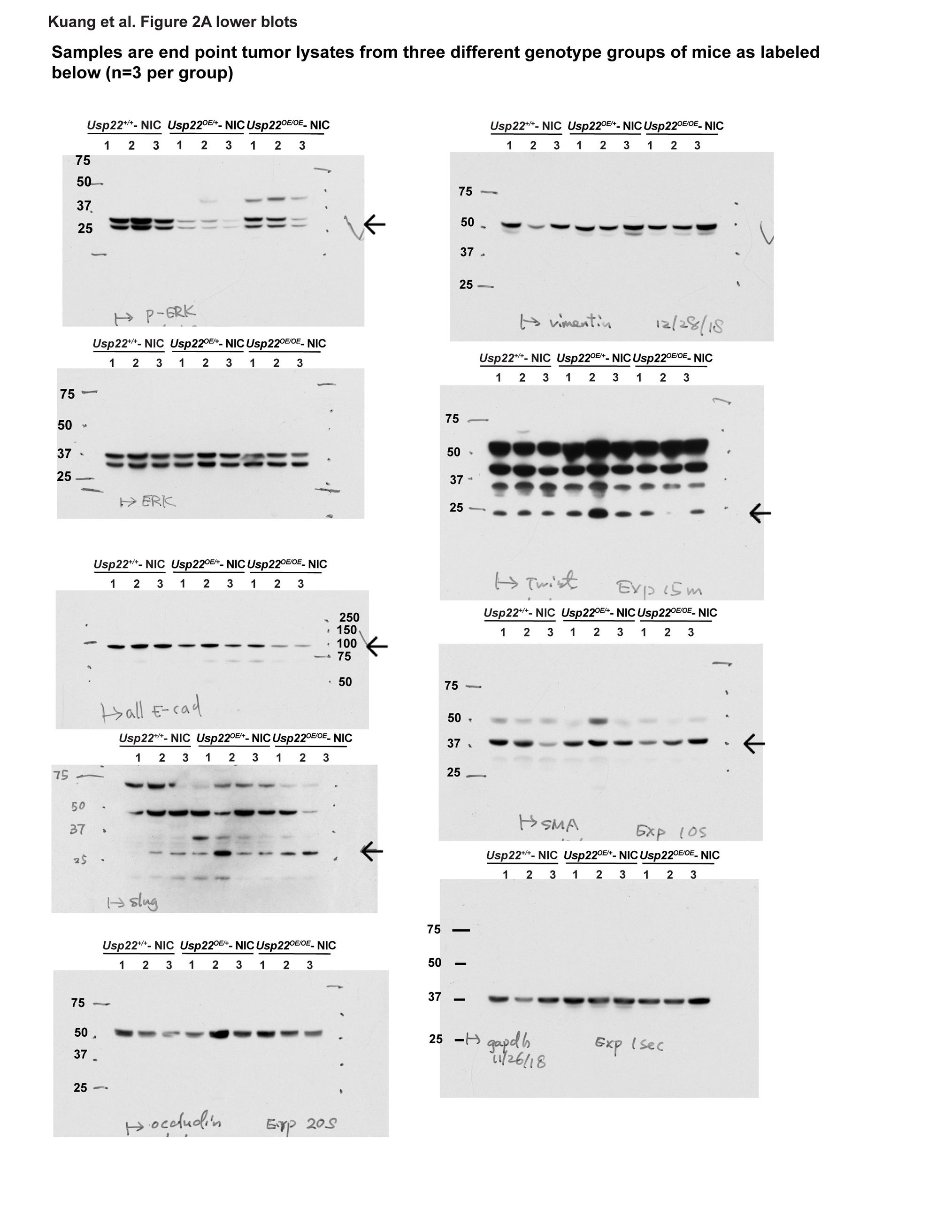

Supplement: S3 Fig — (TIF) [file pone.0290837.s003.tif]

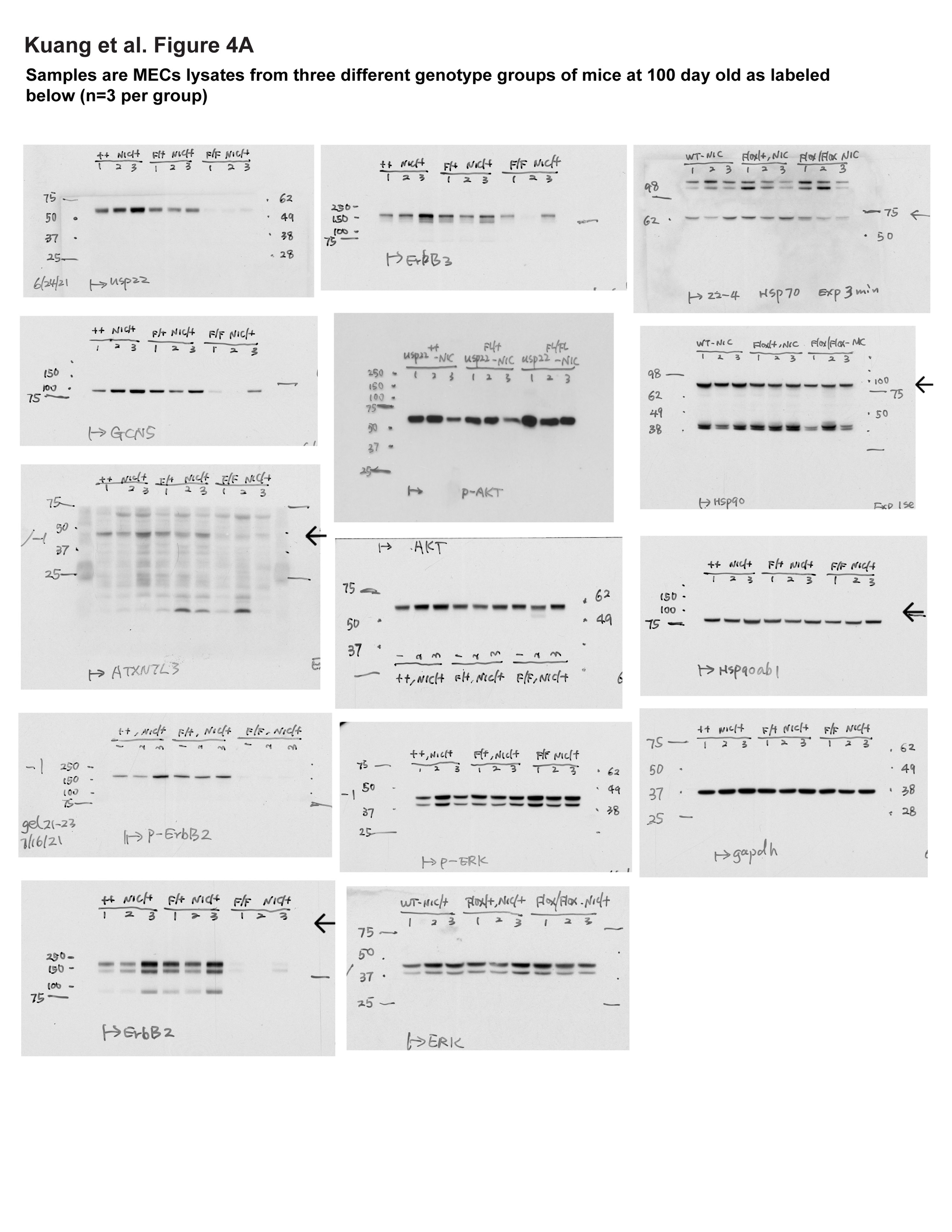

Supplement: S4 Fig — (TIF) [file pone.0290837.s004.tif]

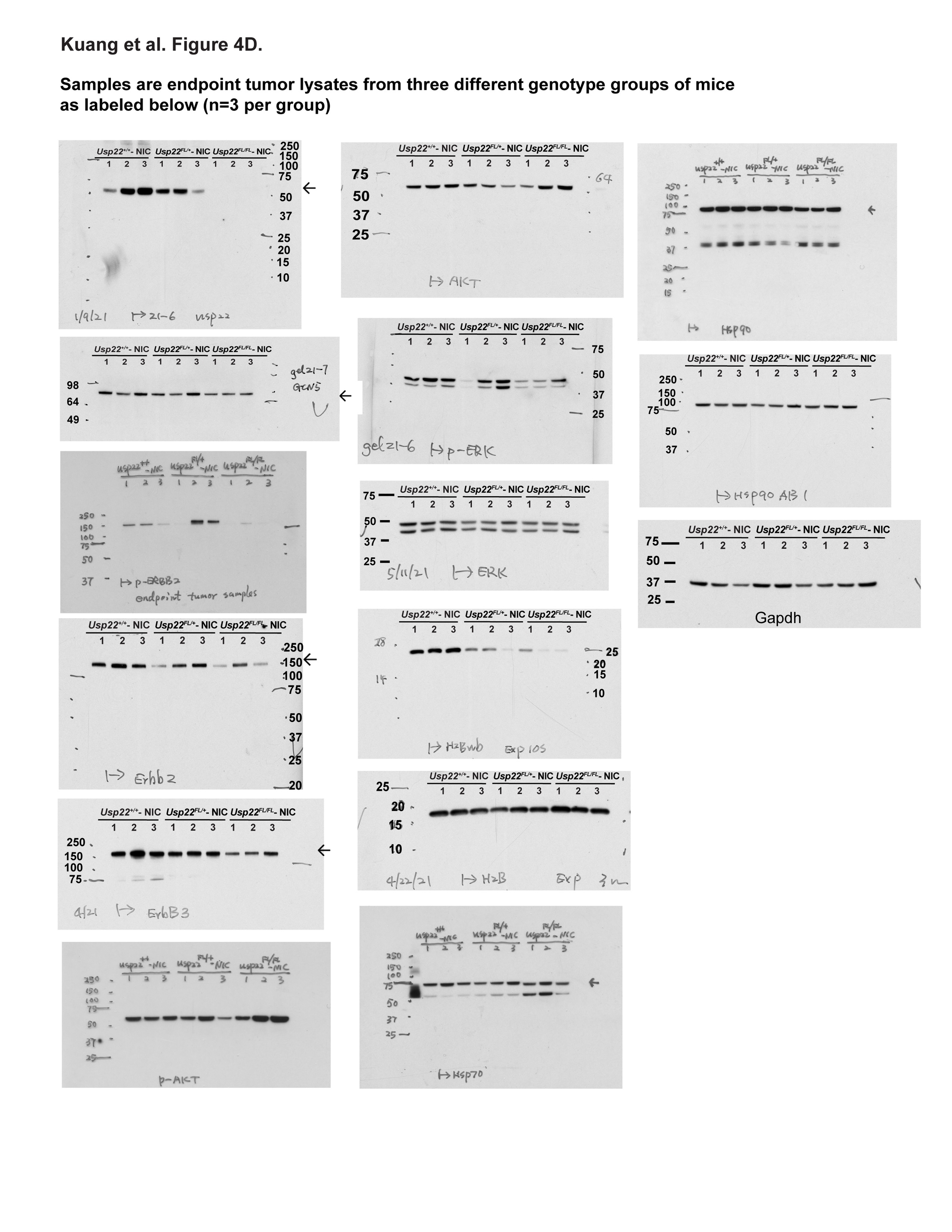

Supplement: S5 Fig — (TIF) [file pone.0290837.s005.tif]

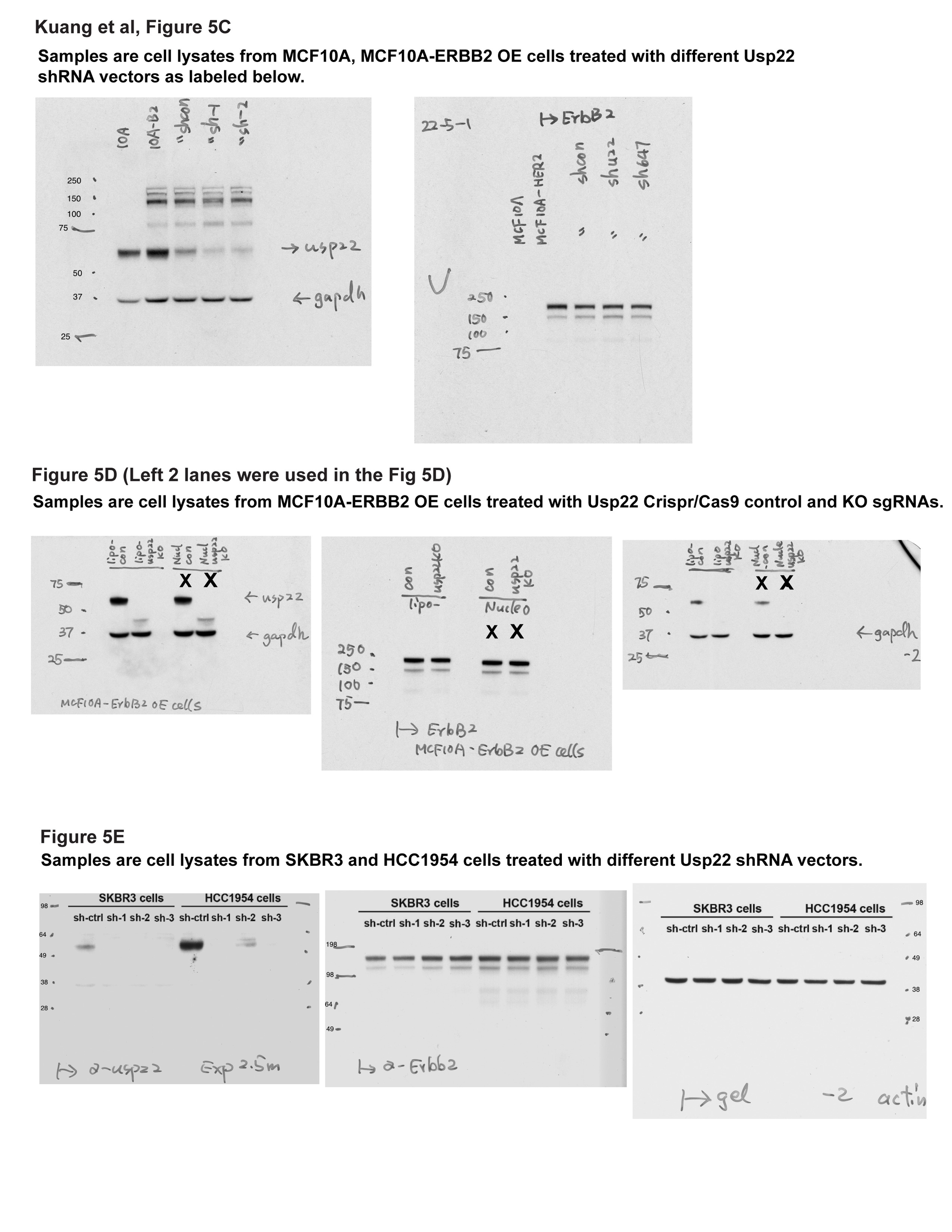

Supplement: S6 Fig — (TIF) [file pone.0290837.s006.tif]

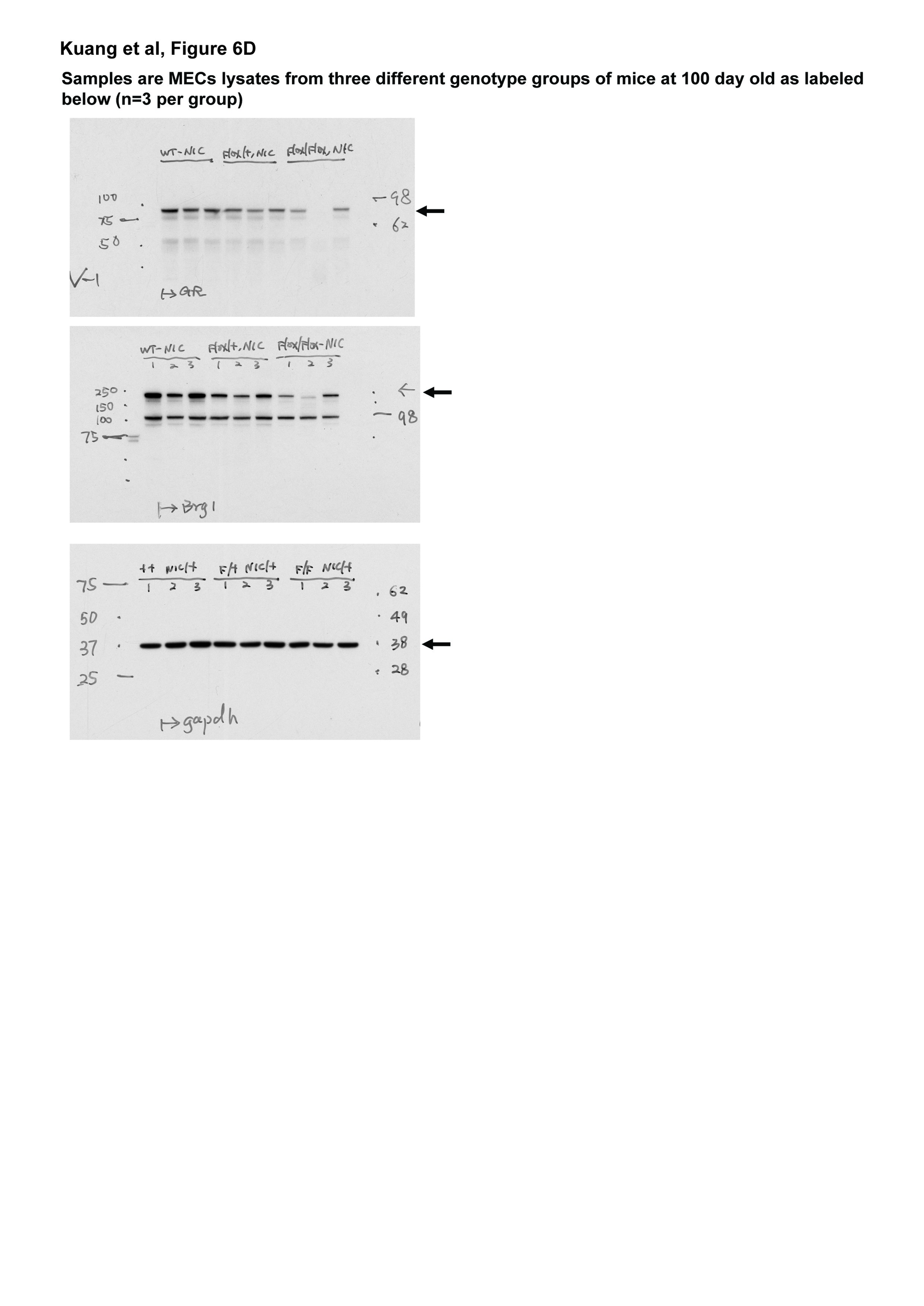

Supplement: S7 Fig — (TIF) [file pone.0290837.s007.tif]

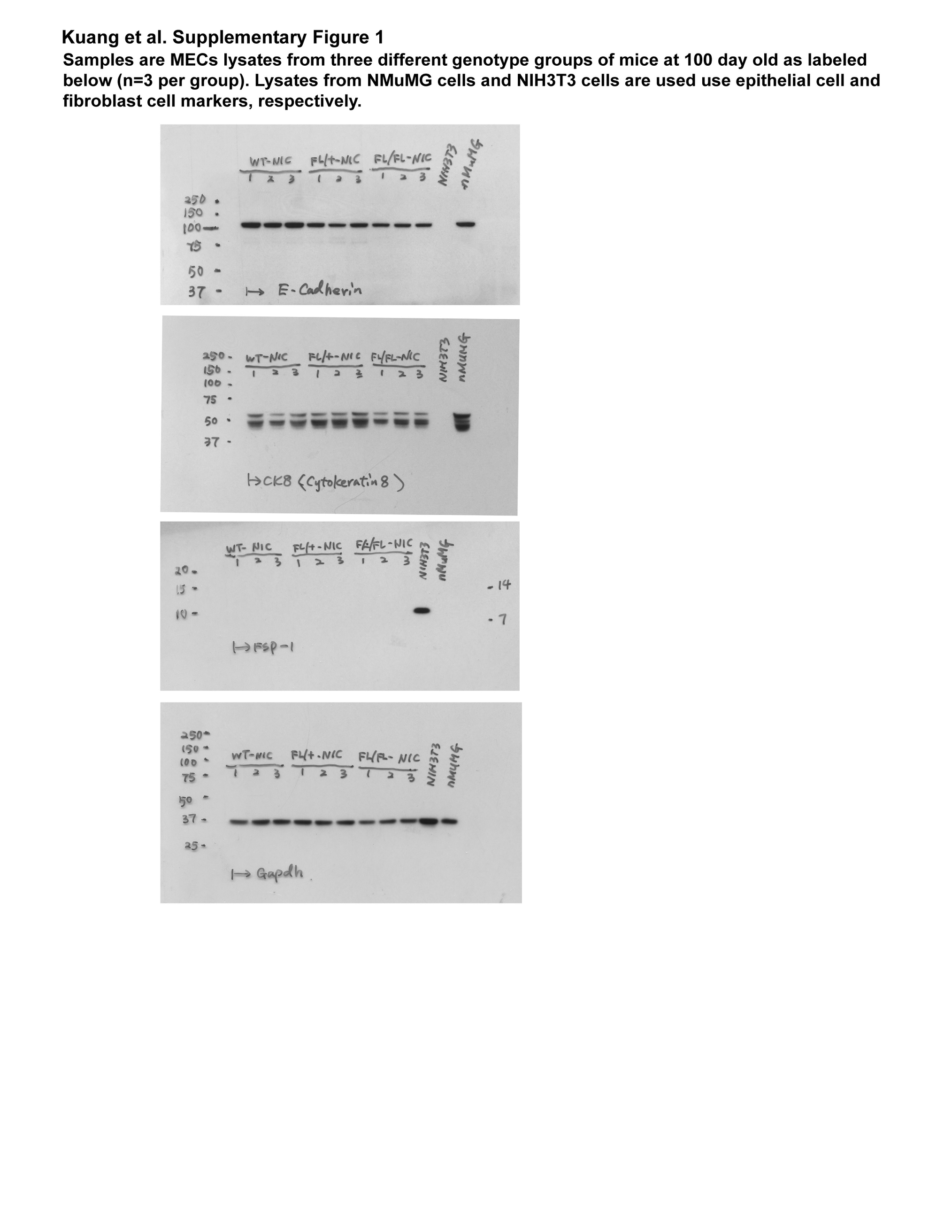

Supplement: S8 Fig — (TIF) [file pone.0290837.s008.tif]
