# Supplementary material for: USP22 overexpression fails to augment tumor formation in MMTV-ERBB2 mice but loss of function impacts MMTV promoter activity
Source: PLoS One. 2024 Jan 18;19(1):e0290837. doi: 10.1371/journal.pone.0290837 (PMC10796002; doi:10.1371/journal.pone.0290837)
Supplement: Attachment — Submitted filename: Review comments _PONE-D-23-12578 .docx [file pone.0290837.s012.docx]

This manuscript reflects work by enthusiasm scientists with a good track record in the field of Usp22 and cancer biology.

Here, the authors identified that modulation Usp22 has different role in MMTV-ERBB2 mediated cancer progression and metastasis.

The present work has been carried out generally with a good experimental standard. The results are presented clearly and the manuscript is well written.

 I have some comments and would be great if they address them.

Figure 1: Could they show the level of Usp22 at mRNA or protein level.

Figure 1: They have high number of mice. It would be better if they could add more mice to the analysis of figure 1.E

Figure 3:  It would be better if they add blot of figure to show the expression of Usp22 in the three groups.

Figure 3: They have high number of mice. It would be better if they could add more mice to the analysis of Ups22 FL/+_NIC group in figure 3.C-E.

Figure 3.b: Please explain which groups that underwent Kaplan-Meier curve and showed significant difference.

Did they observe any change in the organ weight?

Figure 6: Have they investigated blocking the altered hormones in the context of Usp22 modulation?

It will be valuable if the authors elaborate a little bit more the role of the changed in the hormones in the discussion section.
