# Supplementary material for: USP22 overexpression fails to augment tumor formation in MMTV-ERBB2 mice but loss of function impacts MMTV promoter activity
Source: PLoS One. 2024 Jan 18;19(1):e0290837. doi: 10.1371/journal.pone.0290837 (PMC10796002; doi:10.1371/journal.pone.0290837)
Supplement: Attachment — Submitted filename: USP22 overexpression fails to augment tumor formation in MMTV-ERBB2 mice but loss of function impacts MMTV promoter activity.docx [file pone.0290837.s013.docx]

Comment:

This is depth and an interesting study entitled “*USP22 overexpression fails to augment tumor formation in MMTV-ERBB2 mice but loss of function impacts MMTV promoter activity*” and I am convinced that the authors have collected a unique dataset using cutting edge methodology to address the questions. The paper is generally well written and structured. However, in my opinion the paper has some shortcomings regarding the role(s) and mechanism of USP22 still remains questionable and not clear, but the authors have established USP22 overexpression does not accelerate or amplify tumorigenesis in MMTV-NIC 219 mice.

Furthermore, I made additional questions for more in-depth analyses of the data. Key critical points are.

a) Although the role of USP22 in cancer is not clear but it plays important many signal pathways, tumorigenesis and metastatic risk. Can you explain how these studies are different from previously reports about the USP22 and how much it contributes to the basic science?

b) Why have you not used a recently discovered USP22 inhibitor (Morgan *et al.,* 2021) to corroborate your USP22 deletion/knockdown effect on tumorigenesis and metastatic? Do you have any such plan in future?
